# Corticosteroid therapy for critically ill patients with COVID-19: A structured summary of a study protocol for a prospective meta-analysis of randomized trials

**DOI:** 10.1186/s13063-020-04641-3

**Published:** 2020-08-24

**Authors:** Jonathan A. C. Sterne, Janet Diaz, Jesús Villar, Srinivas Murthy, Arthur S. Slutsky, Anders Perner, Peter Jüni, Derek C. Angus, Djillali Annane, Luciano Cesar Pontes Azevedo, Bin Du, Pierre-Francois Dequin, Anthony C. Gordon, Cameron Green, Julian P. T. Higgins, Peter Horby, Martin J. Landray, Giuseppe Lapadula, Amelie Le Gouge, Marie Leclerc, Jelena Savović, Bruno Tomazini, Balasubramanian Venkatesh, Steve Webb, John C. Marshall, for the WHO COVID-19 Clinical Management and Characterization Working Group

**Affiliations:** 1 Population Health Sciences, Bristol Medical School, University of Bristol, Bristol, UK; 2 NIHR Bristol Biomedical Research Centre, Bristol, UK; 3 Clinical Unit, Health Emergencies Programme, World Health Organization, Geneva, Switzerland; 4 Research Unit, Hospital Universitario Dr. Negrin Las Palmas de Gran Canaria, Las Palmas, Spain; 5 CIBER de Enfermedades Respiratorias, Instituto de Salud Carlos III, Madrid, Spain; 6 Department of Pediatrics, University of British Columbia, Vancouver, Canada; 7 Applied Health Research Centre, Li Ka Shing Knowledge Institute of St. Michael’s Hospital, Department of Medicine, University of Toronto, Toronto, Canada; 8 Department of Intensive Care, Rigshospitalet, Copenhagen, Denmark; 9 Department of Critical Care Medicine, University of Pittsburgh School of Medicine, Pittsburgh, PA USA; 10 Department of Intensive Care, Raymond Poincaré Hospital (APHP), School of Medicine Simone Veil, University Paris Saclay –UVSQ, Paris, France; 11 Critical Care and Emergency Medicine, Hospital Sirio Libanês, São Paulo, Brazil; 12 Peking Union Medical College Hospital, Beijing, China; 13 Médecine Intensive - Réanimation, INSERM CIC1415, CHRU de Tours, Tours, France; 14 CRICS-TriGGERSep network, Centre d’Etude des Pathologies Respiratoires, Université de Tours, Tours, France; 15 Division of Anaesthetics, Pain Medicine & Intensive Care, Imperial College London, London, UK; 16 Australian and New Zealand Intensive Care Research Centre, School of Public Health and Preventive Medicine, Monash University, Melbourne, Australia; 17 NIHR Applied Research Collaboration (ARC) West, University Hospitals Bristol and Weston NHS Foundation Trust, Bristol, UK; 18 Nuffield Department of Medicine, University of Oxford, Oxford, UK; 19 Nuffield Department of Population Health, University of Oxford, Oxford, UK; 20 MRC Population Health Research Unit, University of Oxford, Oxford, UK; 21 NIHR Oxford Biomedical Research Centre, Oxford University Hospitals NHS Foundation Trust, Oxford, UK; 22 Division of Infectious Diseases, San Gerardo Hospital, ASST Monza, Monza, Italy; 23 CIC INSERM 1415 - CHRU de Tours, Hôpital Bretonneau, Tours, France; 24 Délégation à la Recherche Clinique et à l’Innovation, CHRU de Tours, Tours, France; 25 George Institute for Global Health, University of New South Wales, Sydney, Australia; 26 Li Ka Shing Knowledge Institute, St. Michael’s Hospital, University of Toronto, Toronto, Canada

**Keywords:** COVID-19, Randomised controlled trial, Systematic Review, Corticosteroid, Dexamethasone, Hydrocortisone, Methylprednisolone, Mortality, Meta-analysis

## Abstract

**Objectives:**

Primary objective: To estimate the effect of corticosteroids compared with usual care or placebo on mortality up to 28 days after randomization. Secondary objectives: To examine whether the effect of corticosteroids compared with usual care or placebo on mortality up to 28 days after randomization varies between subgroups related to treatment characteristics, disease severity at the time of randomization, patient characteristics, or risk of bias. To examine the effect of corticosteroids compared with usual care or placebo on serious adverse events.

**Study design:**

Prospective meta-analysis of randomized controlled trials. Both placebo-controlled and open-label trials are eligible.

**Participants:**

Hospitalised, critically ill patients with suspected or confirmed COVID-19.

**Intervention and comparator:**

Intervention groups will have received therapeutic doses of a steroid (dexamethasone, hydrocortisone or methylprednisolone) with IV or oral administration immediately after randomization.

The comparator groups will have received standard of care or usual care or placebo.

**Main outcome:**

All-cause mortality up to 28 days after randomization.

**Search methods:**

Systematic searching of clinicaltrials.gov, EudraCT, the WHO ISRCTN registry, and the Chinese clinical trials registry. Additionally, research and WHO networks will be asked for relevant trials.

**Risk of bias assessments:**

These will be based on the Cochrane RoB 2 tool, and will use structured information provided by the trial investigators on a form designed for this prospective meta-analysis.

**Summary of findings:**

We will use GRADE to assess the certainty of the evidence.

**Statistical analyses:**

Trial investigators will provide data on the numbers of participants who did and did not experience each outcome according to intervention group, overall and in specified subgroups. We will conduct fixed-effect (primary analysis) and random-effects (Paule-Mandel estimate of heterogeneity and Hartung-Knapp adjustment) meta-analyses. We will quantify inconsistency in effects between trials using I^2^ statistics. Evidence for subgroup effects will be quantified by ratios of odds ratios comparing effects in the subgroups, and corresponding interaction p-values. Comparisons between subgroups defined by trial characteristics will be made using random-effects meta-regression. Comparisons between subgroups defined by patient characteristics will be made by estimating trial-specific ratios of odds ratios comparing intervention effects between subgroups then combining these using random-effects meta-analysis. Steroid interventions will be classified as high or low dose according to whether the dose is greater or less than or equal to 400 mg hydrocortisone per day or equivalent. We will use network meta-analysis methods to make comparisons between the effects of high and low dose steroid interventions (because one trial randomized participants to both low and high dose steroid arms).

**PROSPERO registration number:**

CRD42020197242

**Full protocol:**

The full protocol for this prospective meta-analysis is attached as an additional file, accessible from the Trials website (Additional file 1). To expedite dissemination of this material, the familiar formatting has been eliminated; this Letter serves as a summary of the key elements of the full protocol for the systematic review.

## Supplementary information

**Additional file 1.**

## Data Availability

All data provided by the trials, including summary outcome data overall and in the specified subgroups, will be included in supplementary material of the report of the prospective meta-analysis.

