# Supplementary material for: Corticosteroid therapy for critically ill patients with COVID-19: A structured summary of a study protocol for a prospective meta-analysis of randomized trials
Source: Trials. 2020 Aug 24;21:734. doi: 10.1186/s13063-020-04641-3 (PMC7443535; doi:10.1186/s13063-020-04641-3)

Prospective meta-analysis of randomized trials of corticosteroid therapy for COVID-19

Protocol and analysis plan (version 13), 11 July 2020

1. Introduction

During a pandemic there is a heightened need for rapid reliable information to guide the clinical management of patients with the disease. The early surge in cases creates both a crisis and an opportunity: it amplifies the need for effective treatments, both for the individual patient and to minimize the burden on the health care system, and accelerates the clinical trial process because of the marked increase in the number of cases.

The COVID-19 pandemic has seen multiple trials launched, many of which address similar interventions. In the absence of a mechanism for central harmonization of research activity, these trials proceed with individual criteria for stopping the trial – either because an a priori sample size has been recruited, or because a stopping rule has been met at an interim analysis.

Although individual trials may be under-powered to draw robust conclusions regarding benefit, harm, or futility at their interim or even final analyses, pooling their data might provide sufficient power. One approach could be to have confidential sharing of interim data directly between relevant DMCs. However, the logistics of such an approach would be difficult, especially when there are more than a few ongoing trials.

A prospective meta-analysis (PMA) is a systematic review and meta-analysis of studies that are identified, evaluated and determined to be eligible for the meta-analysis before the relevant results of any of those studies become known. PMA has the potential to provide timely results with maximal precision and minimum risk of bias. Its key feature is that study selection criteria, hypotheses, and analyses are specified before the results of the studies are known. PMAs are most applicable to high priority research questions where limited previous evidence exists and where new studies are expected to emerge.

This document presents a protocol for a prospective meta-analysis of trials of corticosteroid therapy for patients hospitalized with COVID-19. It is based on guidance in Chapter 22 of the Cochrane Handbook for Systematic Reviews of Interventions. It will be registered on the PROSPERO international prospective register of systematic reviews, and published online before outcome data are received.

On 16 June the RECOVERY trial reported rate ratios for 28-day mortality for patients treated with dexamethasone (6 mg/day) of 0.65 (95% CI 0.51 to 0.82) in ventilated patients and 0.80 (0.70 to 0.92) in patients receiving oxygen only. Early drafts of the protocol were written in advance of the release of results from the RECOVERY trial. These included the choice of primary outcome, subgroups to be compared, and approach to analysis. Changes after 16 June that are based on results from RECOVERY are identified in this protocol and will be identified in reports of the results of this PMA.

We hope that this exercise, if successful, will lead to prospective efforts to combine data from trials of the efficacy of other drugs and drug classes in COVID-19 patients, and in any future pandemics.

2. Objectives, outcomes and eligibility

The overall objective of this PMA is to estimate the effect of corticosteroids compared with usual care in hospitalised, critically ill patients with suspected or confirmed COVID-19. The outcome is all-cause mortality up to 28 days after randomization. Shorter-term mortality (e.g., 21 days) will be

acceptable if longer-term mortality is not available. We will also consider combining data on serious adverse events (see sections 6 and 12).

Longer-term mortality (90 days, 180 days) and other outcomes (eg duration of ventilation, length of ICU stay, repeat hospital admissions) will be requested from all trials and will be included as secondary outcomes in a future meta-analysis.

3. Search methods

Trials were identified through systematic searching of clinicaltrials.gov, EudraCT, the WHO ISRCTN registry, and the Chinese clinical trials registry. Additionally, research and WHO networks were asked for relevant trials.

4. Trial eligibility to participate in this prospective meta-analysis

Table 1 at the end of this document summarises details of the trials identified for potential inclusion in this PMA, including their trial registration identifiers and the number of participants recruited to date. All trials exclusively recruited critically ill patients, except for the RECOVERY trial. There were minor variations in definitions of 'critically ill' used to specify each trial's eligibility criteria. The RECOVERY trial recruited both hospitalized and critically ill patients, with the majority being non critically ill. We will request that RECOVERY provides data on mechanically ventilated patients for inclusion in this PMA. Trial protocols will be compared to make final decisions on those for which data pooling appears justifiable, based on recruitment of sufficiently similar patient groups, treatment with similar steroid interventions, and employing similar comparator interventions. All such decisions will be made in advance of sharing of outcome data.

Invitations offering participation in this PMA have been sent to the trial Principal Investigators by the WHO Chief Scientist on behalf of the Clinical Characterization and Management Working Group of the WHO. Participation will be based on this protocol. If further eligible trials are located while the PMA is in progress (but before sharing of outcome data), the PIs of these trials will be sent the protocol and invited to participate. Participation will be confirmed when the trial Principal Investigator indicates their willingness to participate, subject to the procedures described in this protocol. No additional trials will be included after outcome data are shared.

If results from trials eligible for, but not included in this PMA become available before results of the PMA are published, we will conduct and report additional meta-analyses including these results.

5. Risk of bias assessments

We will assess the risk of bias in the overall effect on mortality reported by each trial, based on the Cochrane Risk of Bias Assessment Tool (RoB 2). We will assess the effect of assignment to intervention (the 'intention-to-treat' effect). Risk of bias assessments will be based on the trial protocols and CONSORT flow charts together with the following information, which will be supplied by each trial:

1. Methods used to generate the allocation sequence and conceal randomized allocation;
2. Whether patients and health professionals were blinded to assigned intervention;
3. Methods used to ensure that patients received their allocated intervention;
4. Methods used to measure 28-day mortality.

Risk of bias assessments will be done by independent teams in Bristol and Copenhagen. Disagreements will be resolved through discussion, with consensus assessments reported and analysed.

6. Approach to combined analyses

We will not collect individual-participant data. Trial investigators will provide summary tables showing numbers of participants who did and did not experience each outcome according to intervention group, overall and in the following specified subgroups. For trials with multiple steroid arms, the arms will be combined into a single steroid group provided that the assigned intervention involves starting steroids immediately after randomization. The outcomes will be (1) 28-day mortality and (2) serious adverse events, if these are considered to be sufficiently comparable across trials. Details of the definitions and collection of serious adverse events will be collected in advance of sharing outcome data (see sections 2 and 12).

Subgroups related to treatment or disease severity at the time of randomization:

1. Mechanical ventilation at time of randomization (yes or no);
2. Dexamethasone or prednisolone or hydrocortisone (none of the trials randomized patients to more than one type of corticosteroid);
3. Use of high-dose steroids (equal to or greater than an equivalent of 400 mg hydrocortisone/day, see Table 3);
4. On vasoactive medication at the time of randomization (yes or no).

Subgroups related to patient characteristics (these will not influence a decision to report results):

5. Age (above or below the median age of participants recruited across the trials, calculated based on preliminary communication with trial investigators);
6. Sex.

Risk of bias (see section 5):

7. Low risk of bias, some concerns, high risk of bias;

Post-hoc analysis based on results reported by the RECOVERY trial (this will be reported both including and excluding data from RECOVERY):

8. Symptomatic for >7 days at the time of randomization (yes or no).

We will also compare the effect in critically ill patients, including RECOVERY trial patients who were mechanically ventilated at the time of randomization, with the effect in non-mechanically ventilated patients recruited into the RECOVERY trial.

Trial investigators will also provide summary information on characteristics of patients at the time of randomization and numbers of patients lost to follow up, which will be tabulated and which will provide useful contextual information, but will not be used in analyses.

We will conduct fixed-effect (inverse-variance weighted) and random-effects (with Paule-Mandel estimate of heterogeneity and Hartung-Knapp adjustment) meta-analyses of overall and subgroup effects. The primary analysis will be the fixed-effect meta-analysis of overall mortality. We will quantify inconsistency in effects between trials heterogeneity using I^2 statistics. We will report precise p values and will not use a threshold for statistical significance.

Evidence for subgroup effects will be quantified by ratios of odds ratios comparing effects in the subgroups, and corresponding interaction p-values. Comparisons between subgroups defined by trial characteristics (for example, dexamethasone versus prednisolone versus hydrocortisone, or risk of bias) will be made using random-effects meta-regression ('across-trial' approach). Interpretation of meta-regression results will be cautious because of the potential for confounding by other trial

characteristics. Comparisons between subgroups defined by patient characteristics (for example, age and sex) will be made following recommendations by Fisher et al. (BMJ 2017; 356: j573), by estimating trial-specific ratios of odds ratios comparing intervention effects between subgroups ('within-trial' approach), then combining these using random-effects meta-analysis (using Paule-Mandel and Hartung-Knapp methods). A hybrid approach will be adopted for characteristics (for example, whether mechanically ventilated) that vary between participants in some but not all trials. In this hybrid approach, we will use a within-trial approach restricted to the trials where this is possible, and compare this with an across-trial approach in which effects in subgroups are estimated in separate meta-analyses, and ratios of odds ratios derived from the overall effect in each subgroup. Interpretation will be cautious because of the potential for confounding of across-trial comparisons by other trial characteristics. Some trials randomised participants to both low and high dose steroid arms, while others randomised all participants to a single dose. Therefore, we will use network meta-analysis methods to make comparisons between the effects of high and low dose steroid interventions.

7. Certainty of the evidence

We will use the GRADE approach to rate the certainty of the evidence for the overall effect of steroids across the trials.

8. What will an agreement to participate in this PMA entail?

Trials that agree to participate in this PMA will agree to the following:

a. To share their protocol in advance, and to share aggregated outcome data.

b. To share summary data in a prespecified format. These data will be limited to a small number of agreed upon elements, including confirmation of key aspects of the trial design (Table 1), CONSORT 2010 flow diagram (Figure 1), summary baseline demographics (Table 2) and 2×2 tables (overall and in specified subgroups) for the association of interventions with mortality. Unblinded pooled data will be analysed according to a prespecified analysis protocol. Additional meta-analyses, conducted according to a prespecified protocol, will include results of any relevant trials with results available at the time the analyses are conducted.

c. To agree to a pre-specified process for reporting results to decision-makers and submitting pooled results for publication.

9. Reporting of results

As soon as the analyses are complete, they will be released to:

a. Trial PIs, for sharing as appropriate with research ethics boards overseeing each trial.
b. Each trial sponsor who will have 48 hours to review the results and discuss them with the PIs of the other included trials, prior to releasing them.
c. The Secretary-General of the WHO, or their designate, for sharing with the relevant guideline development committee.

A paper reporting these findings will be prepared and submitted for publication. The paper will be published using an agreed group title. All members of trial steering committees will be included as authors, together with all individuals involved in conducting the PMA. A writing committee including one or more representatives from each trial as well as individuals involved in conducting and reporting the PMA will be established, and its members listed. A list of all contributors to each trial will be provided as supplementary material.

10. Consequences for individual trials

Trial PIs/Steering committees (usually with input from their DMCs) will consider whether to suspend ongoing patient enrolment (on the basis of benefit, harm or futility) from the time of the first communication of the results to the trial Principal Investigator. Subsequent trial management will be at the discretion of each trial PI and his/her trial steering committee, and is likely to be guided by a number of factors including the consistency of the findings across trials and the perceived relevance to the question addressed in their trial. A trial might opt to continue recruitment if the population, the intervention, or the efficacy signal differed substantively from the overall pooled data. A decision to terminate or continue the trial would be communicated to the appropriate REBs and to the study funder, with a detailed rationale for the decision made.

11. Logistics, management and co-ordination

Data will be securely transferred to a central repository at the World Health Organization for statistical analyses.

12. Timelines

Release of results from the RECOVERY trial on 16 June 2020 is likely to have led to treatment of patients assigned to usual care with dexamethasone or other steroids. Because the usual course of treatment is for 7 days after randomization, we will include patients randomized up to and including 9 June 2020. Therefore, 28-day mortality will be ascertained up to and including Monday 6 July 2020. Data will be combined as soon as possible after that date.

Table 1. Trials of corticosteroids in hospitalised patients with COVID-19.

Question marks denote "to be confirmed"; I/E = inclusion/exclusion criteria; MV: = mechanical ventilation.

Clinical Trial	Identifier	Patients recruited on admission to ICU?	Phase	Planned N	N randomized to June 14th	Experimental intervention (only corticosteroid interventions are listed)	Control	Mortality	Contacts and location
Efficacy and Safety of Corticosteroids in COVID-19	NCT04273321	No?	N/A	400	86 (terminated; submitted to a journal)	Methylprednisolone (1 mg/kg/day x 7 days)	No intervention I/E do not mention antivirals	30 days	Bing Sun & Shi Huannzhong, China
COVID-19-associated ARDS Treated with Dexamethasone: Alliance Covid-19 Brasil III (CoDEX)	NCT04327401	Yes	III	350	282	Dexamethasone (20 mg/day x 5 days then 10 mg/day x 5 days) + standard treatment	Standard treatment Antivirals are allowed	28 days	Luciano Cesar Pontes Azevedo & Bruno Tomazini, Brazil
Efficacy of Dexamethasone Treatment for Patients with ARDS Caused by COVID-19	NCT04325061	Yes	IV	200	19	Dexamethasone (20 mg/day x 5 days and 10 mg/day x 5 days) + standard treatment	Standard treatment Antivirals are allowed	60 days 28 days also possible	Jesus Villar & Arthur Slutsky, Spain
Randomized, Embedded, Multifactorial Adaptive Platform Trial for Community-Acquired Pneumonia (REMAP-CAP)	NCT02735707	Yes	IV	N/A	420	Hydrocortisone 200mg/day x 7 days + standard treatment or Hydrocortisone 400mg/day x 7 days + standard treatment	Standard treatment. Antivirals are allowed.	21 days<br>90 days	Cameron Green, Steve Webb, Colin McArthur, Marc Bonten, Lennie Derde, John Marshall, Derek Angus, global study
A randomized, open-label study to evaluate the efficacy and safety of low-dose corticosteroids in hospitalized patients with novel coronavirus pneumonia	ChiCTR 2000029656	No	0	100	?	Standard treatment + methylprednisolone (low-dose)	Standard treatment I/E do not mention antivirals	No	Ronghui Du & Li Li China
The clinical value of corticosteroid therapy timing in the treatment of novel coronavirus pneumonia (COVID-19)	ChiCTR20000 30481	Yes	IV	200	Discontinued for lack of enrolment ?	Early corticosteroid (?) intervention Mid-late corticosteroid (?) intervention	No corticosteroid	21 days	Chen Zhenshun, Wuhan, China. No answer received from these investigators

Clinical Trial	Identifier	Patients recruited on admission to ICU?	Phase	Planned N	N randomized to June 14th	Experimental intervention (only corticosteroid interventions are listed)	Control	Mortality	Contacts and location
Glucocorticoid Therapy for Novel Coronavirus Critically Ill Patients with Severe Acute Respiratory Failure (Steroids-SARI)	NCT04244591	Yes	II/III	80	47 (unfinished; submitted to CCM journal)	Standard of care + methylprednisolone (80 mg/day x 5 days) All patients received antivirals	Standard of care All patients received antivirals	Yes	Bin Du & Li Weng, China
Randomised Evaluation of COVID-19 Therapy (RECOVERY)	NCT04381936	No	II/III	N/A	6425, of whom 1007 were mechanically ventilated	Dexamethasone (6 mg/day x 10 days)	Standard treatment Antivirals are allowed in the dexamethasone arm (to be verified)	28 days	Peter Horby, Martin Landray, Wei Shen Lim, Kenneth Baillie, Richard Haynes, Ed Juszczak & Thomas Jaki, United Kingdom
Low dose hydrocortisone in patients with COVID-19 and severe hypoxia – the COVID STEROID Trial	NCT04348305	No	III	1,000	29	Hydrocortisone (200 mg/day x 7 days)	Sodium chloride solution (placebo) (antivirals registered before randomization)	90 days	Anders Perner, Marie Warrer, & Maj-Brit Nørregaard Kjær, Denmark
Community-Acquired Pneumonia: Evaluation of Corticosteroids (CAPE_COD)	NCT02517489	Yes	III	290 (?)	149	Hydrocortisone (200 mg/day x 4 or 7 days + 100 mg/day x 2 or 4 days + 50 mg/day x 2 or 3 days) + standard treatment of severe community-acquired pneumonia	Placebo + standard treatment of severe community-acquired pneumonia	28 days	Pierre-Francois Dequin & Marie Leclerc, France
Dexamethasone and Oxygen Support Strategies in ICU patients with Covid-19 Pneumonia (COVIDICUS)	NCT04344730	Yes	N/A	550	14 (All patients received antivirals)	Dexamethasone (20 mg/day x 10 days) + (conventional oxygen OR CPAP OR HFNO OR mechanical ventilation)	Placebo + (conventional oxygen OR CPAP OR HFNO OR mechanical ventilation)	60 days	Jean Francois Timsit, Lila Boudama, Veronique Deiler & Sylia Zmihi, France
10 trials (8 trials in ICU) (excluding REMAP-CAP and RECOVERY)					626				

Table 2: Summary descriptive data to be provided by each trial

	Steroid	No steroid
PCR-confirmed SARS-COV-2 infection (N, %)		
Mechanical ventilation at time of randomization (if applicable) (N, %)		
On vasoactive medication at the time of randomization (N, %)		
Female sex (N, %)		
Median (IQR) age (years)		
Treated with remdesevir at the time of randomization (N, %)		
Treated with lopinavir/ritonavir at the time of randomization (N, %)		
Treated with favipravir at the time of randomization (N, %)		
Treated with hydroxychloroquine at the time of randomization (N, %)		
Treated with azithromycin at the time of randomization (N, %)		
Treated with convalescent plasma at the time of randomization (N, %)		

Table 3. Classification of steroid interventions in each trial, based on a cut off of equivalent of Hydrocortisone 400 mg/day, Dexamethasone 15 mg/day)*

Clinical Trial	Country	Identifier	Dose(s)	Classification
**Dexamethasone trials**				
CoDEX	Brazil	NCT04327401	20 mg/day x 5 days then 10 mg/day x 5 days	High
Efficacy of Dexamethasone for ARDS Caused by COVID-19	Spain	NCT04325061	20 mg/day x 5 days then 10 mg/day x 5 days	High
RECOVERY	UK	NCT04381936	6 mg/day x 10 days	Low
COVIDICUS	France	NCT04344730	20 mg/day x 10 days	High
**Hydrocortisone trials**				
REMAP-CAP	Global	NCT02735707	(1) 200mg/day x 7 days<br>(2) 400mg/day x 7 days	(1) Low<br>(2) High
COVID STEROID	Denmark	NCT04348305	200 mg/day x 7 days	Low
CAPE_COD	France	NCT02517489	(1) 200 mg/day x 4 or 7 days<br>(2) 100 mg/day x 2 or 4 days<br>(3) 50 mg/day x 2 or 3 days	(1) Low<br>(2) Low<br>(3) Low
**Methylprednisolone trials**				
Efficacy and Safety of Corticosteroids in COVID-19	China	NCT04273321	1 mg/kg/day x 7 days	High
Steroids-SARI	China	NCT04244591	80 mg/day x 5 days	High

*Based on Djillali Annane, Stephen M. Pastores, Bram Rochwerg, Wiebke Arlt, Robert A. Balk, Albertus Beishuizen, Josef Briegel, Joseph Carcillo, Mirjam Christ-Crain, Mark S. Cooper, Paul E. Marik, Gianfranco Umberto Meduri, Keith M. Olsen, Sophia Rodgers, James A. Russell & Greet Van den Berghe. Guidelines for the diagnosis and management of critical illness-related corticosteroid insufficiency (CIRCI) in critically ill patients (Part I): Society of Critical Care Medicine (SCCM) and European Society of Intensive Care Medicine (ESICM) 2017. Intensive Care Medicine 2017; 43: 1751–1763. https://link.springer.com/article/10.1007/s00134-017-4919-5

Figure 1. CONSORT 2010 Flow Diagram

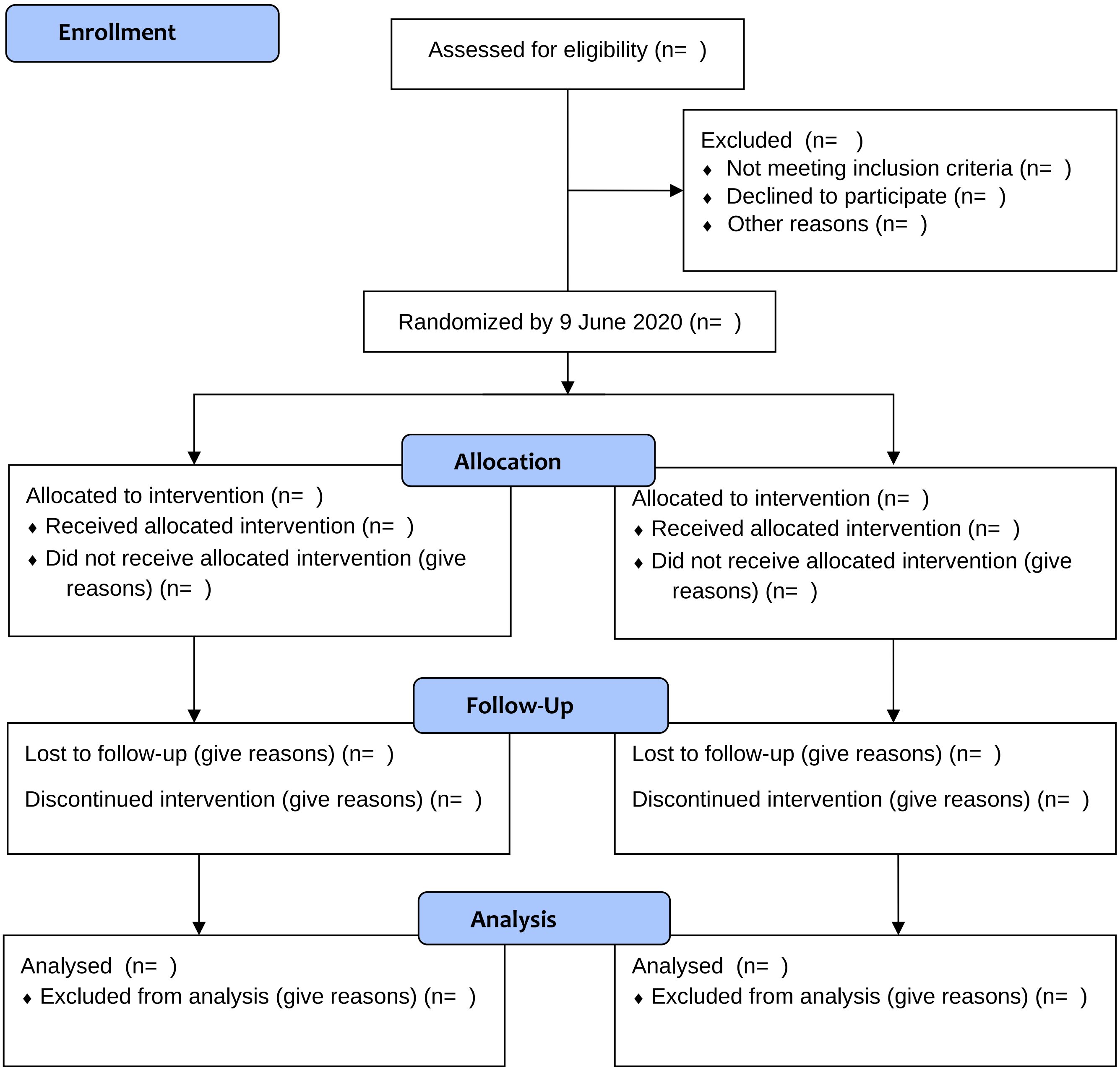

Supplement: Supplementary file 1 — Additional file 1. [file 13063_2020_4641_MOESM1_ESM.pdf]
